# S-RNase-based self-incompatibility in angiosperms: Degradation, condensation, and evolution

**DOI:** 10.1093/plphys/kiaf360

**Published:** 2025-08-14

**Authors:** Yongbiao Xue

**Affiliations:** Laboratory of Advanced Breeding Technology, Institute of Genetics and Developmental Biology, Chinese Academy of Sciences, Beijing 100101, China; University of Chinese Academy of Sciences, Beijing 100049, China

## Abstract

The S-RNase-based self-incompatibility (SI) system is a key mechanism in angiosperms that safeguards against self-pollination, thereby promoting outcrossing and genetic diversity. Governed by a single multiallelic *S*-locus, this system is regulated by pistil-expressed S-RNases and pollen-expressed *S*-locus F-box (SLF) proteins. In cross-pollination, SLFs assemble into Skp1/Cullin1//F-box (SCF) ubiquitin ligase complexes. These complexes selectively recognize non-self S-RNases and target them for proteasomal degradation, allowing pollen tube growth to proceed unimpeded and fertilize the host ovule. In self-pollination, S-RNases capable of escaping degradation by self SCF complex undergo phase separation, forming cytoplasmic condensates that disrupt the cytoskeleton and redox homeostasis, ultimately triggering programmed cell death. Considered the ancestral SI system, S-RNase-based incompatibility likely emerged through the evolutionary linkage of ancestral S-RNase and SLF genes into a proto-*S-*locus. Other SI systems in angiosperms are hypothesized to have evolved secondarily via the loss of ancestral components within this evolutionary framework. Future research priorities include elucidating the molecular basis of SLF-mediated recognition of diverse S-RNases, unraveling the complex genetic architecture of the *S*-locus, and identifying novel SI mechanisms in understudied angiosperm lineages. This review underscores SI's molecular sophistication and evolutionary plasticity, highlighting its fundamental role in plant reproduction and its relevance to agricultural breeding strategies.

## Introduction

Organisms maintain genetic diversity within their populations through outcrossing, an evolutionary strategy that employs strikingly divergent mechanisms across the animal and plant kingdoms (Hiscock et al. 1996). Approximately 80% of angiosperm species possess bisexual flowers (Proctor et al. 1996). While this hermaphroditic system enhances reproductive efficiency, the physical proximity of pistils (housing the ovules) and stamens (containing the pollen) inevitably increases the risk of self-pollination and thus inbreeding. To mitigate the evolutionary pressure of inbreeding depression (reduced population fitness), plants with bisexual flower have developed multifaceted antiselfing strategies, including dichogamy (temporal separation of sexual organ maturation), herkogamy (spatial separation of sexual organs), and self-incompatibility (SI) (de Nettancourt 2001; Barrett 2002).

**Figure 1.**
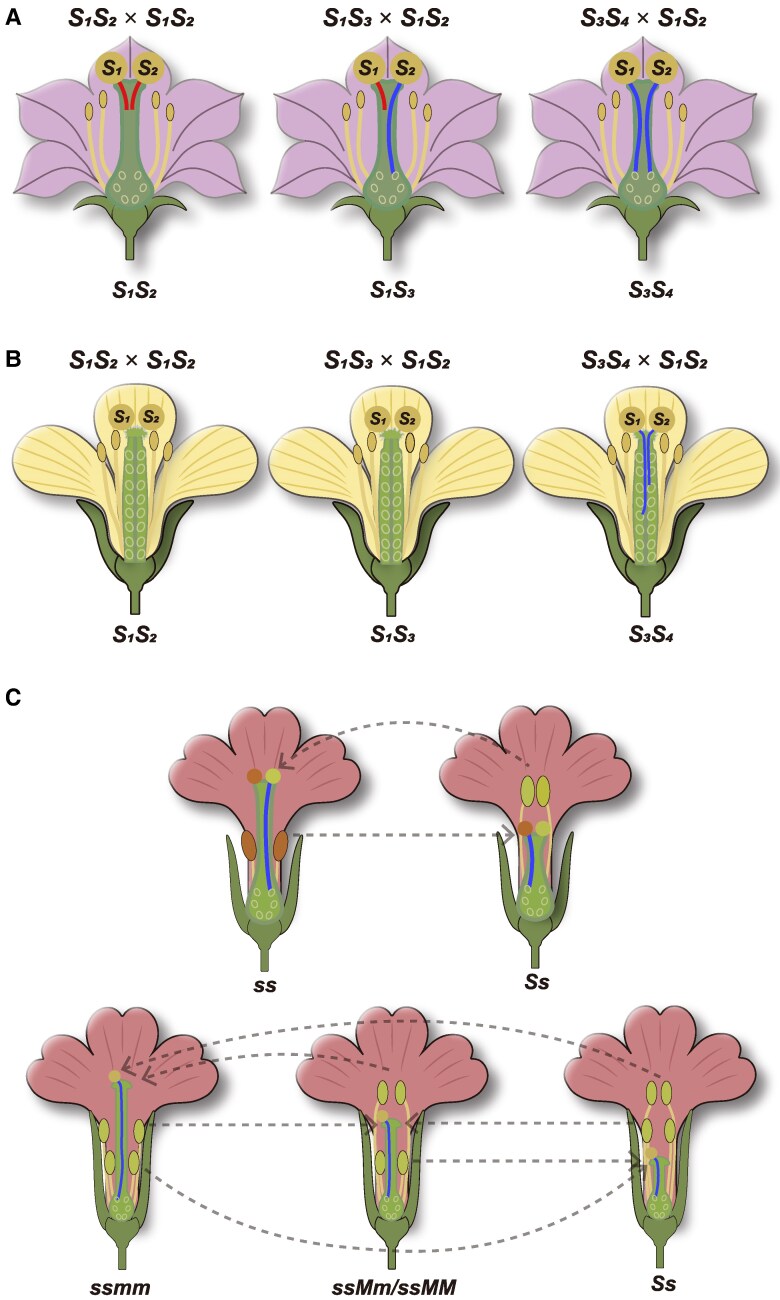
Genetic control of SI in angiosperms. **A)** GSI. GSI is determined by the pollen (gametophyte) *S* genotype. Pollen tubes grow normally only when the pollen *S* genotype does not match the pistil *S* genotype. Left: Self-pollination, where the pollen *S* genotype (*S*_1_*S*_2_) matches the pistil *S* genotype (*S*_1_*S*_2_), resulting in pollen tube growth inhibition. Middle: Semi cross-pollination, where the pollen *S* genotype (*S*_1_*S*_2_) half matches the pistil *S* genotype (*S*_1_*S*_3_), and about half of the pollen tubes grow normally. Right: Cross-pollination, where the pollen *S* genotype (*S*_1_*S*_2_) does not match the pistil *S* genotype (*S*_3_*S*_4_), allowing all pollen tubes to grow normally. **B)** SSI. SSI is determined by the maternal (sporophyte) genotype. Pollen tube growth occurs when the pollen and stigma express different SI specificities. In fact, due to the presence of dominance/recessiveness and codominance among *S* haplotypes, the genetic control of SSI and crossing combinations are highly complex. This diagram simplifies the *S* haplotypes as exhibiting complete dominance, with the dominance hierarchy being *S*_1_ > *S*_2_ > *S*_3_ > *S*_4_. Left: Self-pollination, pollen tube growth is inhibited due to the specifically expressed stigma and pollen genes residing within the same dominant haplotype *S*_1_. Middle: Cross-pollination, pollen tube growth is inhibited due to the specifically expressed stigma and pollen genes residing within the same dominant haplotype *S*_1_. Right: Cross-pollination, pollen tube growth proceeds normally due to the specifically expressed stigma gene from dominant haplotype *S*_3_ and pollen gene from dominant haplotype *S*_1_ residing on different haplotypes. **C)** HSI. HSI species exhibit 2 (distyly) or 3 (tristyly) floral morphs. Distyly comprises 2 morphs: long-styled morph (long style and short stamens, genotype *ss*) and the short-styled morph (short style and long stamens, genotype *Ss*). Tristyly includes 3 morphs: long-styled morph (long style and short stamens), mid-styled morph (medium-length style and stamens of both long and short lengths), and short-styled morph (short style and long stamens), with genotypes *ssmm*, *ssMm/ssMM*, and *Ss*, respectively. Dashed lines indicate compatible crosses.

SI is the genetic mechanism in which fertile hermaphroditic plants fail to produce viable seeds following self-pollination (de Nettancourt 2001; Franklin-Tong 2008; Nasrallah 2023). This mechanism is prevalent in ∼50% of angiosperm species (Goring et al. 2023). SI is a prezygotic reproductive barrier employed by hermaphroditic plants to avoid self-fertilization and promote outcrossing. The occurrence of SI begins when pistil cells distinguish between self- and non-self-pollen. This recognition leads to the failure of self-pollen tubes to germinate or elongate normally down the style of the pistil toward the ovule, resulting in their failure to complete the fertilization process (de Nettancourt 2001; Franklin-Tong 2008; Nasrallah 2023). SI systems are categorized into 2 major types: homomorphic SI and heteromorphic SI (HSI). The homomorphic SI systems can be classified into gametophytic SI (GSI) and sporophytic SI (SSI) (Fig. 1, A and B). In GSI, pollen tube growth inhibition takes place within the transmitting tract of the style. Pollen tube growth is permitted only when the haploid genotype of the pollen differs from that of the pistil. In SSI, pollen tube growth inhibition occurs at the stigma surface. Pollen tubes can only grow when the pollen and stigma express different SI specificities. The HSI system is very different from the above pollen phenotype-based classifications, as it involves a distinct floral morphology-dependent SI system (Shang et al. 2025). HSI species exhibit 2 (distyly) or 3 (tristyly) floral morphs (Fig. 1C). Distyly includes 2 floral types, “thrum” (short pistil and long stamens) and “pin” (long pistil and short stamens). Tristyly includes a 3rd morph, the mid-styled flower, characterized by a medium-length pistil and stamens of both long and short lengths. SI occurs to prevent within-morph fertilization, while cross-compatibility happens between different morphs (Eckert and Barrett 1993; Kivastik et al. 2025).

Many homomorphic SI systems are governed by a multiallelic *S*-locus comprising distinct tightly linked functional units with each *S* haplotype encoding a pistil *S* determinant expressed specifically in female reproductive tissues and a pollen *S* determinant in male gametophyte (Takayama and Isogai 2005; Zhang et al. 2009). In grass species, although SI is controlled by a 2-locus (*S* and *Z*) system (Lundqvist 1954; Hayman 1956), the *S* and *Z* loci also contain linked genes for pistil- and pollen-specific components (Yang et al. 2008; Herridge et al. 2022; Wang et al. 2022; Rohner et al. 2023).

**Figure 2.**
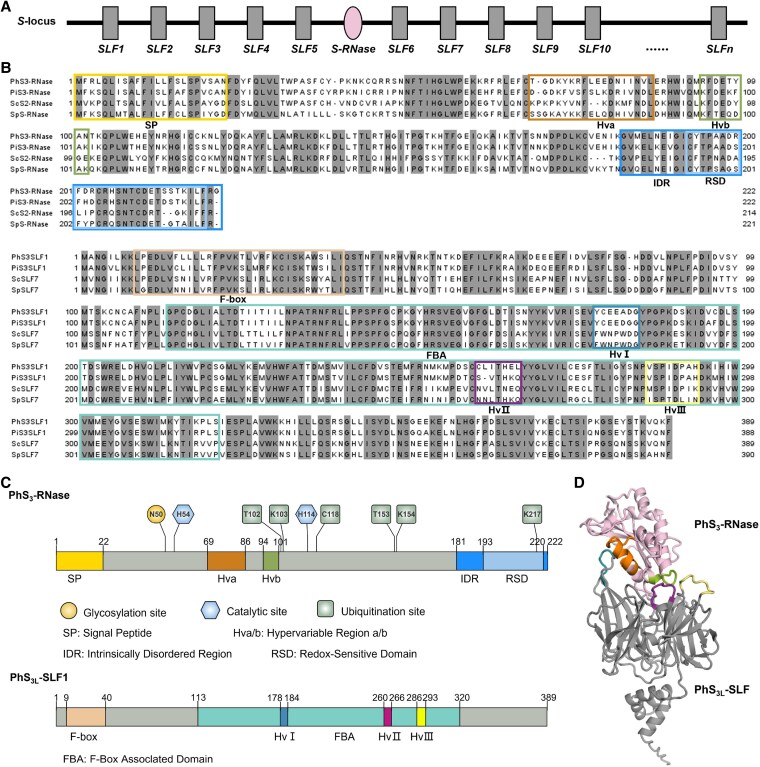
Genomic and structural features of *S-RNases* and *SLFs* in Solanaceae. **A)** Genomic organization of the *S*-locus, showing the clustered arrangement of multiple *SLF* genes and a single *S-RNase* gene. Note that the order of genes is schematic and does not represent their actual genomic positions. **B)** Multiple sequence alignments of 4 representative S-RNases (top) and 4 SLF proteins (bottom) from *Petunia* and *Solanum*. Conserved structural domains are highlighted with colored boxes: signal peptide (SP), hypervariable regions a/b (Hva/Hvb), IDR, and RSD in S-RNases; and F-box, FBA domain, and 3 hypervariable regions (HvI to HvIII) in SLFs. **C)** Domain architectures of PhS_3_-RNase and PhS_3L_-SLF1, with color-coded regions corresponding to boxed domains in **B)**. In addition, functional residues—including the catalytic histidines, a glycosylation site, and multiple ubiquitination sites—are indicated along the sequence. **D)** Predicted protein complex structure of PhS_3_-RNase and PhS_3L_-SLF1 based on AlphaFold3. Structural domains are color-coded as in **B** and **C)**, illustrating the spatial configuration of potential recognition surfaces.

Since molecular mechanisms underlying SI are diverse among different families, recent studies have recognized 8 types of SI (Zhao et al. 2022; Wang et al. 2025). The most extensively distributed mechanism is type-1, also known as Solanaceae-type or S-RNase-based SI, found in Plantaginaceae, Solanaceae, Rosaceae, Rutaceae, Cactaceae, and Primulaceae, which is controlled by a single pistil *S S-RNase* and a cluster of pollen *S*-locus F-box (*SLFs*) ranging from 9 to 37 genes (Fig. 2A) (Anderson et al. 1986; McClure et al. 1989; Xue et al. 1996; Lai et al. 2002; Ushijima et al. 2003; Sijacic et al. 2004; Qiao et al. 2004a; Sassa et al. 2007; Liang et al. 2020; Ramanauskas and Igić 2021; Ramanauskas et al. 2025). By contrast, the other 7 types involve different classes of protein. Type-2 SI (Brassicaceae) is determined by stigma *S-locus receptor kinase* (*SRK*) and pollen *S-locus protein 11* (*SP11*)/*S-locus cystine-rich* (*SCR*) (Schopfer et al. 1999; Suzuki et al. 1999; Takasaki et al. 2000; Takayama et al. 2000), Type-3 (Papaveraceae) by *Papaver rhoeas stigma S* (*PrsS*) and *P. rhoeas pollen S* (*PrpS*) (Foote et al. 1994; Wheeler et al. 2009), Type-4 SI (*Primula*) by *cytochrome P450* (*CYP*), *GLOBOSA2* (*GLO2*), *Kelch repeat F-box* (*KFB*), *conserved cysteine motif* (*CCM*), and *Pumilio-like RNA-binding protein* (*PUM*) (Huu et al. 2016, 2020; Li et al. 2016), Type-5 SI (*Turnera*) by *Turnera subulate S-protein homolog 1* (*TsSPH1*), *T. subulate YUCCA6* (*TsYUC6*), and *T. subulate BAHD* (*TsBAHD*) (Shore et al. 2019), Type-6 SI (Poaceae) by *Hordeum pistil S-specific10* (*HPS10*-*S*/*Z*) and *DUF247I*/*II*-*S*/*Z* (*domain of unknown function 247*) (Kakeda et al. 2008; Lian et al. 2021; Herridge et al. 2022; Wang et al. 2022; Rohner et al. 2023), Type-7 SI (Linaceae) by *Linum tenue thrum style-specific gene1* (*LtTSS1*) and *L. tenue WD repeat-containing protein 44* (*LtWDR44*) (Gutiérrez-Valencia et al. 2022), and Type-8 SI (Oleaceae) by *Gibberellin-acid-2-oxidase* (*GA2oxis*) (Castric et al. 2024; Raimondeau et al. 2024) have been discovered.

This review explains how recent advances in the study of Type-1 SI mechanisms have discovered the molecular roles of pistil *S* S-RNase and pollen *S* SLF proteins. By integrating established models—including the S-RNase degradation and condensation model and the S-RNase compartmentalization model—this article shows the regulatory network underlying these models. From an evolutionary perspective, S-RNase-based SI represents the ancestral state in angiosperms (Xue et al. 1996; Igic and Kohn 2001; Steinbachs and Holsinger 2002; Zhao et al. 2022), with other SI types arising through secondary evolutionary events following the loss of ancestral *S-*locus components. Finally, this article outlines future research directions, emphasizing challenges in understanding SI polymorphism maintenance, molecular coevolution dynamics, and applications in agricultural breeding.

## Discoveries and structural features of S-RNase and SLF proteins

### Discovery of S-RNase

The identification of *S-RNase* as a key factor in plant SI was initiated by isolating a pistil-specific glycoprotein in *Nicotiana alata* that cosegregated with *S-*haplotypes (Bredemeijer and Blaas 1981; Anderson et al. 1986) (Table 1). Sequence analyses revealed its similarity to fungal T2-type ribonucleases and its in vitro nuclease activity, led to its designation as *S-RNase* (McClure et al. 1989) (Table 1). Gain- and loss-of-function assays in *Petunia* and *Nicotiana* demonstrated that *S-RNase* determines whether pollen carrying a matching *S-*haplotype is accepted or rejected, without affecting pollen SI function (Lee et al. 1994; Murfett et al. 1994). The relationship between *S-RNase* and SI was later validated in a broad range of species, including *Solanum tuberosum* (Solanaceae), *Antirrhinum hispanicum* (Plantaginaceae), *Malus* × *domestica* and *Pyrus serotina* (Rosaceae), *Citrus* (Rutaceae), *Schlumbergera truncata* (Cactaceae), and *Lysimachia monelli* (Primulaceae) (Sassa et al. 1996; Xue et al. 1996; Liang et al. 2017; Ye et al. 2018; Ramanauskas and Igić 2021; Ramanauskas et al. 2025) (Table 1). Sequence-related genes have also been cloned from other plant species, such as *Camellia sinensis* (Theaceae), though their functions remain experimentally unconfirmed (Zhang et al. 2016).

**Table 1.** Pistil factors involved in S-RNase-based SI

Genes	Species	Functions	References
*S*_2_ *-RNase*	*N. alata*	Pistil *S* specificity	Anderson et al. (1986)
*S-RNase*s	*N. alata*	Pistil *S* specificity	McClure et al. (1989)
	*A. hispanicum*	Pistil *S* specificity	Xue et al. (1996)
	*Malus* × *domestica*	Pistil *S* specificity	Sassa et al. (1996)
	*Py. serotina*	Pistil *S* specificity	
	*Schlumbergera truncata*	Pistil *S* specificity	Ramanauskas and Igić (2021)
	*Citrus*	Pistil *S* specificity	Liang et al. (2020)
	*Lysimachia monelli*	Pistil *S* specificity	Ramanauskas et al. (2025)
*HT-B*	*N. alata*	Self-pollen rejection	McClure et al. (1999)
		Compartmentalization	Goldraij et al. (2006)
	*S. tuberosum*	Self-pollen rejection	Lee et al. (2023)
	*Petunia*	Self-pollen rejection	Puerta et al. (2009)
	*Pe. hybrida*	Condensation	Tian et al. (2024)
	*Lycopersicon*	Self-pollen rejection	Kondo et al. (2002)
*Trxh*	*N. alata*	S-RNase activity	Torres-Rodríguez et al. (2020)
	*Pe. hybrida*	Condensation	Tian et al. (2024)
*120K*	*Nicotiana*	Self-pollen rejection	Hancock et al. (2005)
		Compartmentalization	Goldraij et al. (2006)
*StEP*	*N. alata*	HT-B protection and PCD	Jiménez-Durán et al. (2012)
			Cruz-Zamora et al. (2020)

### Discovery of SLF

Following the molecular characterization of the pistil component *S-RNase*, efforts identified the pollen determinant of SI: an *S*-haplotype-specific gene encoding an F-box protein tightly linked to the *AhS*_2_*-RNase* in *A. hispanicum* (Lai et al. 2002) (Table 2). This gene, termed *AhSLF-S*_2_, exhibited pollen-specific expression and strict cosegregation with the corresponding *S-*haplotype, forming part of a single *S-*locus. *SLF* homologs were subsequently identified across multiple families, including Solanaceae and Rosaceae (Sijacic et al. 2004; Sassa et al. 2007; Kubo et al. 2010) (Table 2). In the subfamily *Maloideae* of Rosaceae, *SLF* is named *S-locus F-box brothers* (*SFBB*) (Sassa et al. 2007) (Table 2). By contrast, the *Prunoideae* is believed to contain 2 types of *SLF* genes: one type is homologous to *SLFs* from other species and is termed *SLF-like* (*SLFL*), while the other is unique to this subfamily and is called *S-haplotype-specific F-box* (*SFB*) (Ushijima et al. 2003; Matsumoto et al. 2008) (Table 2). Multiple *SLF* paralogs were found to be encoded within the *S-*locus in several species, suggesting that pistil-expressed *S-RNase* and multiple pollen-expressed *SLF*s function cooperatively to determine SI specificity (Zhou et al. 2003; Kubo et al. 2010; Fujii et al. 2016; Sun et al. 2018; Vieira et al. 2019). Genetic evidence for the role of *SLF*s in SI primarily relies on competitive interaction, a phenomenon in which the heterozygous diploid pollen produced by tetraploid plants can cause breakdown of SI (Crane and Lewis 1942; Lewis 1947). Based on this principle, studies have verified the function of *SLFs* by transforming them into *Petunia hybrida*, *Pe. inflata,* or *Citrus* to detect whether they can disrupt SI (Sijacic et al. 2004; Qiao et al. 2004a; Kubo et al. 2010; Cao et al. 2024) (Table 2). By contrast, there is some controversy regarding the function of the *SFB* genes. As *SFB* mutations and premature termination were found in self-compatible plants, it is speculated that *SFB* mutations lead to the loss of pollen-side function (Ushijima et al. 2004). Nevertheless, uncertainty persists regarding whether these mutations underlie self-compatibility (SC). Additionally, due to the limited availability of transgenic technology in Rosaceae plants, verifying the function of SFB through reverse genetics is impossible. Despite this, researchers have recently demonstrated that the transgenes of Rosaceae *SLFL*, *SFBB*, and *SFB* can also cause breakdown of SI of *Pe*. *hybrida* through competitive interaction, indicating that *SFB* and *SLF*/*SLFL*/*SFBB* have similar functions (Zhao et al. 2022).

**Table 2.** Pollen factors involved in S-RNase-based SI

Genes	Species	Functions	References
*AhSLF-S*_2_	*A. hispanicum*	Pollen *S* specificity	Lai et al. (2002)
		Pollen *S* specificity	Qiao et al. (2004a)
*PiSLF-S*_2_	*Pe. inflata*	Pollen *S* specificity	Sijacic et al. (2004)
*SLFs*	*Petunia*	Pollen *S* specificity	Kubo et al. (2010)
*S*_6_ *-SLF7a*	*Citrus grandis*	Pollen *S* specificity	Cao et al. (2024)
*SFBs*	*Prunus dulcis*	Pollen *S* specificity	Ushijima et al. (2003)
*SFBBs*	*Malus* × *domestica*	Pollen *S* specificity	Sassa et al. (2007)
	*Py. pyrifolia*	Pollen *S* specificity	
*SSK1*	*A. hispanicum*	SCF^SLF^ formation	Huang et al. (2006)
	*Pe. hybrida*	SCF^SLF^ formation	Zhao et al. (2010)
	*Pe. inflata*	SCF^SLF^ formation	Sun and Kao (2018)
	*Py. bretschneideri*	SCF^SLF^ formation	Xu et al. (2013)
	*Malus* × *domestica*	SCF^SLF^ formation	Yuan et al. (2014)
	*C. grandis*	SCF^SLF^ formation	Cao et al. (2024)
*CUL1-like*	*A. hispanicum*	SCF^SLF^ formation	Qiao et al. (2004b)
*CUL1-P*	*Pe. hybrida*	SCF^SLF^ formation	Kubo et al. (2016)
*CUL1*	*Py. bretschneideri*	SCF^SLF^ formation	Xu et al. (2013)
	*C. grandis*	SCF^SLF^ formation	Cao et al. (2024)
*eEF1A*	*S. chacoense*	Bind of S-RNase to actin	Soulard et al. (2014)
*MVG*	*Malus* × *domestica*	Actin severing	Yang et al. (2018)
*Actin1*	*Py. bretschneideri*	Actin polymerization	Chen et al. (2018)
*PbrPLDδ1*			
*PLC*	*Pyrus*	Ca^2+^ influx	Qu et al. (2017)
*CBL5*	*Malus* × *domestica*	Ca^2+^ concentration gradient	Gu et al. (2015)
*ABCF*	*Malus* × *domestica*	S-RNase transportation	Meng et al. (2014b)
*PPA5*	*Py. bretschneideri*	*PME44* repression	Tang et al. (2023)
*PME44*		Tip growth of pollen tubes	
*SIPP*	*Nicotiana alata*	Mitochondria permeability	García-Valencia et al. (2017)
*CLP*	*Pe. hybrida*	PCD	Zakharova et al. (2024)

### Structural features of S-RNase

As expected for a classical secreted protein, the S-RNase primary translational product contains an N-terminal signal peptide that directs the protein to the extracellular matrix (ECM) of the stylar transmitting tissue, where it accumulates in a mature form lacking the signal peptide sequence (Fig. 2, B and C; Anderson et al. 1986). Following secretion, S-RNases from various haplotypes are taken up into pollen tubes in a nonselective manner (Luu et al. 2000). While the exact mechanism remains unclear, current evidence supports 2 potential entry pathways: endocytosis as demonstrated in tobacco (Goldraij et al. 2006; Roldán et al. 2012) and ABC transporter-mediated uptake, as shown in apple (Meng et al. 2014a). Although the colocalization of S-RNase and Golgi marker was incomplete, both the microtubule and Golgi vesicle systems are suggested to be required for in vitro S-RNase internalization in apple (Meng et al. 2014b).

Structural analysis of S-RNase sequences has revealed a common architecture, including 5 conserved domains (C1 to C5) and 2 hypervariable regions (Hva and Hvb) (Fig. 2, B and C; Ioerger et al. 1991). Despite extensive polymorphism, this domain organization is remarkably conserved across Solanaceae, Rosaceae, Plantaginaceae, and Rutaceae. Allelic specificity has been attributed to residues within the hypervariable regions, as shown by mutational analyses in *S. chacoense* (Matton et al. 1997). Crystallographic studies in *Nicotiana* and *Pyrus* confirmed that hypervariable regions are surface-exposed, consistent with a role in allele-specific recognition (Ida et al. 2001; Matsuura et al. 2001). The conserved C2 and C3 regions resemble the catalytic domains of fungal T2 RNase and contain histidine residues (H54 and H114 of S_3_-RNase; Fig. 2C) essential for RNase activity. Mutation of these residues abolishes self-pollen rejection, suggesting that cytotoxicity relies on ribonuclease function (Huang et al. 1994). However, this enzymatic activity may not be strictly necessary. Heat-inactivated S-RNases can still inhibit pollen tube growth (Gray et al. 1991), and grafting experiments suggest that rRNA degradation may occur downstream of SI signaling, rather than acting as the primary trigger of this signaling (Lush and Clarke 1997).

In *Petunia*, cytoplasmic condensates formed by S-RNases through liquid–liquid phase separation (LLPS) have been observed specifically in self-pollen tubes (Tian et al. 2024). This process is mediated by an intrinsically disordered region (IDR), and deletion of the IDR from PhS_3L_-RNase markedly impairs condensate formation and results in a self-compatible phenotype (Tian et al. 2024). These findings suggest that IDR-mediated LLPS is essential for triggering the SI response. Additionally, the SI response is associated with changes in cellular redox state, with reactive oxygen species (ROS) contributing to signaling cascades (Wang et al. 2010; Serrano et al. 2015). A redox-sensitive domain (RSD) within the IDR enhances responsiveness to redox changes and promotes condensate stability, potentially sustaining SI signaling and self-pollen rejection (Tian et al. 2024).

S-RNases also contain 1 to 5 N-linked glycosylation sites, which vary in number and location among different *S-*haplotypes (Oxley et al. 1996; Ishimizu et al. 1999). In *Pe. inflata*, an N29D substitution that eliminates the sole predicted N-glycosylation site of S_3_-RNase does not impair its ability to mediate SI (Fig. 2C; Karunanandaa et al. 1994). By contrast, in *S. chacoense*, the loss of a glycosylation site in the C2 region results in rejection of multiple pollen haplotypes, suggesting its potential role in maintaining recognition fidelity (Soulard et al. 2013). Glycosylation may also influence the S-RNase threshold required to inhibit pollen tube growth. In *S. chacoense*, a threshold effect has been observed, and reduced glycosylation correlates with lower thresholds (Qin et al. 2001; Liu et al. 2008), though the underlying mechanisms remain to be clarified.

In *Petunia*, S-RNase degradation is finely regulated through site-specific ubiquitination at 6 residues, occurring preferentially during cross-pollination to control its cytotoxicity (Zhao et al. 2021). In this system, non-self S-RNases are polyubiquitinated via K48 linkages by Skp1/Cullin1//F-box (SCF) complexes and degraded by the 26S proteasome, facilitating cross-pollen compatibility (Qiao et al. 2004b; Hua and Kao 2008, 2006; Zhang et al. 2009; Kubo et al. 2010; Liu et al. 2014; Zhao et al. 2021). Specifically, PhS_3_-RNase is ubiquitinated at T102 and K103 (Region I), T153, K154, and K217 (Region II), and C118 (Region III), with Region I modified under all pollination conditions and Regions II and III targeted specifically during cross-pollination (Fig. 2C). Functional studies identify Region II as the primary regulatory site, with Regions I and III playing secondary roles. These findings reveal a residue-specific and hierarchical mechanism for S-RNase control through the ubiquitin–proteasome system (UPS).

### Recognition between S-RNases and SLFs

Physical interactions between pollen-expressed SLFs and pistil-expressed S-RNases form the molecular basis for self-/non-self-recognition in S-RNase-based SI systems. To explain how a finite set of SLFs can recognize a large and diverse repertoire of S-RNases, a “collaborative non-self-recognition” model has been proposed based on transgenic and interaction studies in *Petunia* (Kubo et al. 2010). This model posits that multiple SLFs of low polymorphism function collectively to recognize a diverse set of non-self S-RNases, forming a combinatorial recognition system in which 16 to 20 *SLF* genes, tightly linked to their cognate *S-RNase*, each target one or more non-self S-RNases. This configuration allows for the collective degradation of all non-self *S-*haplotypes while sparing the self S-RNase.

In *Antirrhinum*, biochemical assays demonstrate that the C-terminal region of AhSLF interacts with both self and non-self S-RNases (Qiao et al. 2004b). SLFs exhibit stronger binding to non-self S-RNases than to self S-RNases, reinforcing the role of SLF–S-RNase binding dynamics in allelic recognition (Hua et al. 2007; Kubo et al. 2010; Zhao et al. 2021). Studies in the Rosaceae family have similarly demonstrated direct interactions between SLF proteins and S-RNases. Experimental approaches such as immunogold labeling suggest that these interactions occur within the cytoplasm of pollen tube cells (Luu et al. 2000; Boivin et al. 2014; Liu et al. 2014).

Interaction specificity has been mapped to distinct structural regions within both protein classes. In S-RNases, the hypervariable regions Hva and Hvb are sufficient for interaction with SLFs, as shown by yeast 2-hybrid analysis (Li et al. 2017). There are 3 functional domains (FD1 to FD3) in PiSLF2, with FD2 mediating interaction with S-RNases, while FD1 and FD3 negatively regulate interactions, particularly with self S-RNases. These findings suggest that PiSLF alleles fine-tune S-RNase recognition to somehow control pollen tube fate (Hua et al. 2007). Electrostatic surface potential analysis has revealed that self S-RNases and SLFs tend to share similar surface charge distributions, whereas non-self-interactions are characterized by opposing electrostatic profiles (Li et al. 2017). A positively selected residue, E293, in PhS_3L_-SLF1 was found to alter electrostatic compatibility and shift *S-*haplotype specificity, supporting an “electrostatic recognition” model (Li et al. 2017). However, the lack of experimentally determined high-resolution structures has historically limited the mechanistic interpretation of these interactions. Recent advances using AlphaFold3 (Abramson et al. 2024) have provided high-confidence structural predictions for key S-RNase–SLF pairs (interface predicted template modeling score [iptm] = 0.8; predicted template modeling score [ptm] = 0.86). For example, models of PhS_3_-RNase and PhS_3L_-SLF1 reveal that the hypervariable regions Hva and Hvb of the S-RNase, along with highly variable loop regions HvI to HvIII of the SLF, are positioned at the predicted interaction interface (Fig. 2D). These structural insights provide a mechanistic framework for prior biochemical findings and could guide future studies seeking to elucidate the molecular determinants of allele-specific recognition in S-RNase-based SI.

### Mechanisms of cross-compatibility and SI

#### Models of S-RNase-based SI system

The elucidation of pollen-side incompatibility factors has enabled the development of mechanistic models to explain S-RNase-based SI. As the complexity of this system has become increasingly evident, the need for a coherent conceptual framework to reconcile conflicting observations and guide future research has emerged.

The earliest explanatory framework was the receptor model, which proposed that self S-RNases are selectively imported into pollen tubes while non-self S-RNases are excluded and therefore unable to exert cytotoxicity (Thompson and Kirch 1992). However, subsequent studies demonstrated that both self and non-self S-RNases are internalized into pollen tubes (Luu et al. 2000; Goldraij et al. 2006). In addition, SLF proteins were shown to be cytosolic F-box proteins rather than membrane-bound receptors, leading to widespread rejection of the receptor-based hypothesis (Liu et al. 2014).

An alternative explanation is provided by the inhibitor model, which suggests that SLFs contain distinct motifs for recognizing and inhibiting the catalytic activity of non-self S-RNases, thereby neutralizing their toxicity. By contrast, interaction with self S-RNases fails to inhibit RNase activity, allowing cytotoxicity to proceed (Kao and McCubbin 1996; Kao and Tsukamoto 2004). To account for allelic interactions, a revised version of this model proposed that SLFs form tetrameric complexes capable of recognizing self S-RNases and shielding them from a general inhibitor. In heterozygous pollen, tetramer formation is disrupted, allowing inhibiting both self and non-self S-RNases (Luu et al. 2001). Despite its conceptual appeal, no biochemical evidence has confirmed the existence of SLF tetramers, and this model remains speculative.

#### The degradation and condensation model

**Figure 3.**
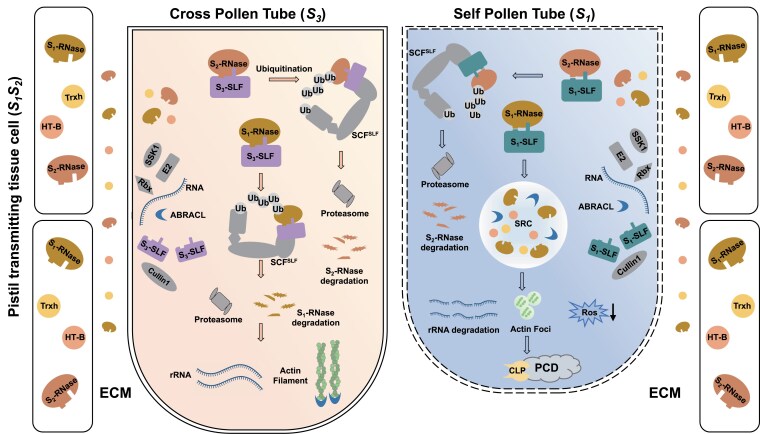
The degradation and condensation model describes the fate of S-RNases in pollen tubes at early stages of pollination in *Petunia*. Both S_1_- and S_2_-RNases are taken up from the ECM of the stylar transmitting tissue. Left: During cross-pollination, S_1_- and S_2_-RNases are recognized as non-self by S_3_-SLF proteins and targeted for polyubiquitination and degradation via the 26S proteasome, allowing normal pollen tube growth. Right: During self-pollination, the SLF complex fails to recognize the self S-RNase (S_1_-RNase), resulting in its cytoplasmic accumulation. Formation of biomolecular condensates by these S-RNases initiates a cascade of downstream events, including RNA degradation, disruption of actin dynamics, depletion of intracellular Ca^2+^, and suppression of ROS production, ultimately culminating in PCD. SRC: S-RNase Condensate.

Accumulating evidence has led to broad acceptance of the S-RNase degradation model (Fig. 3), in which SI is regulated through the UPS. In this model, SLFs, as key components of SCF-type E3 ubiquitin ligase complexes (SCF^SLF^), specifically recognize and polyubiquitinate non-self S-RNases, targeting them for degradation via the 26S proteasome. This process ensures that compatible pollen tubes, which contain non-self S-RNases, effectively remove these toxic molecules to promote fertilization. Direct biochemical validation of this model has been obtained through isolation of active SCF^SLF^ complexes exhibiting E3 ligase activity (Entani et al. 2014), while mutagenesis of lysine residues on S-RNase impairs ubiquitination and subsequent degradation (Hua and Kao 2008; Zhao et al. 2021). Notably, the UPS-mediated degradation of S-RNase occurs exclusively in compatible pollen tubes (Qiao et al. 2004b; Liu et al. 2014; Zhao et al. 2021), consistent with the notion that only non-self S-RNases are targeted for removal, while self S-RNases evade degradation and remain cytotoxic.

This model also provides a mechanistic explanation for competitive interactions during pollination. For instance, in a cross between an *S*_1_*S*_2_ recipient and an *S*_1_*S*_3_ donor, *S*_1_ and *S*_3_ pollen tubes both internalize self and non-self S-RNases, all of which are cytotoxic by default. In the *S*_3_ pollen tube, both S_1_- and S_2_-RNases are recognized as non-self and targeted for ubiquitination by SCF^SLF^ complexes and are subsequently degraded via the 26S proteasome, allowing successful cross-fertilization. By contrast, in the *S*_1_ pollen tube, only S_2_-RNase is degraded, while self S_1_-RNase remains cytotoxic due to the inability of the SCF^SLF^ complex to ubiquitinate it, thereby triggering the SI response.

A critical discovery supporting this model was the identification of SLF-interacting proteins, which are essential components of the SCF^SLF^ ubiquitin ligase complex (Table 2). In *Antirrhinum,* AhSLF has been shown to interact in vivo with a Cullin1-like protein, a core component of the canonical SCF complex (Qiao et al. 2004b). Further studies demonstrated that silencing the *pollen-specific isoform of Cullin1* (*CUL1-P*) via RNA interference results in the loss of pollen compatibility in *Pe. hybrida*. The presence of functionally redundant *CUL1* homologs across species suggests that the role of *CUL1* in maintaining compatibility is evolutionarily conserved (Kubo et al. 2016; Sun et al. 2023). Additionally, SLF-interacting SKP1-like1 (SSK1), a pollen-specific protein, links SLFs to the CUL1 scaffold, forming a functional SCF^SLF^ complex (Huang et al. 2006; Zhao et al. 2010; Xu et al. 2013). In *Petunia*, PhSSK1 physically associates with CUL1-P, and directly interacts with multiple SLFs to facilitate the assembly of the SCF^SLF^ ubiquitin ligase complex. A similar interaction is observed in *Antirrhinum*, where AhSSK1 mediates SLF recruitment to a CUL1-based complex, highlighting a conserved role in SCF complex formation (Huang et al. 2006). Disrupting pollen-specific homologs such as PhSSK1 and PiSSK1 results in the complete loss of compatibility, further solidifying their importance for successful cross-pollination (Zhao et al. 2010; Sun and Kao 2018). These studies also ruled out the involvement of other SCF-like complexes mediated by S-RNase binding protein1 during cross-pollination (Sun and Kao 2018). Similar interactions have also been observed in species from the Rosaceae and Rutaceae (Xu et al. 2013; Yuan et al. 2014; Cao et al. 2024).

Expanding upon the degradation model, the condensation model has been proposed to further explain the cytotoxicity of self S-RNases in *Petunia* (Fig. 3; Tian et al. 2024). Cytological and biochemical evidence indicate that, alongside S-RNases, the small asparagine-rich protein (high top-band [HT-B]) and thioredoxin Type h (Trxh), are taken up into both self- and non-self-pollen tubes. In *Solanum* and *Petunia*, mutations or silencing of *HT-B* correlate with a breakdown of SI, further supporting its role in this process (Kondo et al. 2002 ; Puerta et al. 2009) (Table 1). Notably, in *S. tuberosum*, knockout of *HT-B* alone using CRISPR (Clustered Regularly Interspaced Short Palindromic Repeats)-Cas9 (CRISPR-associated protein 9) yielded few/no seeds, while *HT-B* and *S-RNase* double knockout produced up to 3 times more seeds than *S-RNase*-only knockout, showing their synergistic effect on SI (Lee et al. 2023) (Table 1).

In compatible tubes, SCF^SLF^-mediated degradation prevents S-RNase accumulation and condensate formation. RNA integrity is maintained, actin-binding proteins such as ABRA C-terminal like remain available, and cytoskeletal organization supports normal pollen tube growth. By contrast, for incompatible self-pollen tubes, S-RNases escape SLF recognition and accumulate in the cytoplasm. Through LLPS, these S-RNases form cytoplasmic condensates. PhHT-B and PhTrxh facilitate the stabilization of these condensates under reducing conditions (Table 1). This condensate formation plays a central role in disrupting cellular homeostasis, which promotes the sequestration of actin-binding proteins such as PhABRACL into SRCs and reduces the polymerization activity of PhABRACL in vitro and contributes to the formation of actin foci, ultimately leading to cytoskeletal disorganization and growth arrest (Tian et al. 2024).

The collapse of cytoskeletal integrity is a key step toward triggering programmed cell death (PCD) in incompatible pollen tubes. Supporting this, in *Petunia*, caspase-like protease (CLP) activity increases significantly within several hours of self-pollination (Zakharova et al. 2021, 2024). Exogenous application of cytokinin (CK) inhibits pollen tube growth, a process linked to CK-induced cytoplasmic acidification, and this acidification further enhances CLP activity (Zakharova et al. 2024). Conversely, treatment with the actin-depolymerizing agent Latrunculin B reduces CLP activity in incompatible tubes while elevating activity in compatible ones (Zakharova et al. 2024). These findings indicate that CK-induced cytoplasmic acidification and cytoskeletal status promotes CLP activation and triggers PCD in self-incompatible pollen tubes.

#### The compartmentalization model

**Figure 4.**
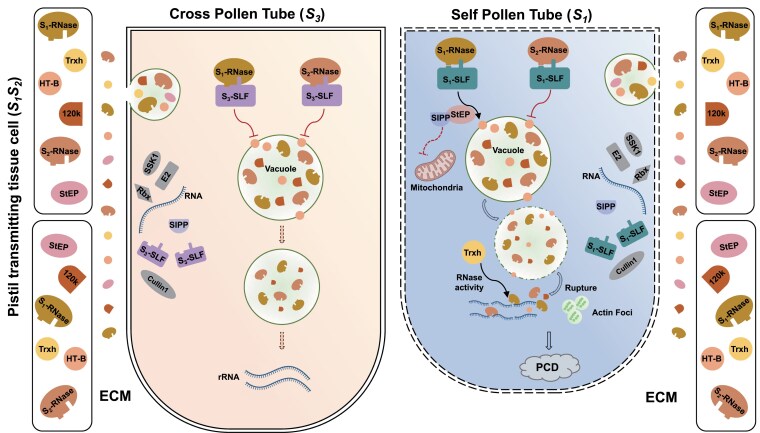
The compartmentalization model highlights the subcellular trafficking of S-RNases and the regulatory role of HT-B in *Nicotiana*. Upon uptake, both self and non-self S-RNases are initially sequestered into vacuoles. In compatible pollen tubes, S_3_-SLFs recognize S_1_- and S_2_-RNases as non-self in the cytosol. Simultaneously, SLF-mediated destabilization of HT-B helps maintain vacuolar integrity, thereby preventing the release of residual S-RNases. In incompatible pollen tubes, S_1_-SLFs fail to recognize the self S_1_-RNase, leading to stabilization of HT-B, vacuole rupture, and release of active S-RNases into the cytosol. These S-RNases induce RNA degradation, actin filament depolymerization, Ca^2+^ depletion, reduction of ROS levels, and PCD, ultimately resulting in pollen tube arrest.

In *Nicotiana*, the compartmentalization model emerged from the observation that S-RNases, although taken up into pollen tubes, predominantly localize to vacuoles, with only a minor fraction detected in the cytosol (Fig. 4; Goldraij et al. 2006). This intracellular distribution, together with the identification of HT-B, led to the proposal that S-RNase trafficking and localization influence SI outcomes (McClure et al. 1999; Kondo et al. 2002). According to this model, interaction with non-self S-RNases promotes HT-B degradation, resulting in vacuolar sequestration and inactivation of the RNases. Supporting this idea, HT-B is highly expressed in pistils and may primarily be involved in postrecognition processes (Goldraij et al. 2006) (Table 1). The fact that loss of HT-B results in SC despite normal S-RNase accumulation suggests that HT-B acts beyond the initial S-RNase uptake, possibly regulating their intracellular fate and activity (McClure et al. 1999). In addition to HT-B, other pistil-side components also appear to contribute to the modulation of S-RNase cytotoxicity (Table 1). The 120-kDa glycoprotein (120K), which is abundant in the stylar ECM, enters pollen tubes after pollination and is required for the rejection of self-pollen in *N. alata*. Its direct in vitro interaction with S-RNases supports a role in reinforcing the SI response, likely by stabilizing or facilitating RNase function within the pollen tube cytoplasm (Cruz-Garcia et al. 2005; Hancock et al. 2005).

By contrast, SLF-mediated recognition of self S-RNases stabilizes HT-B, preventing its degradation and thereby triggering vacuolar rupture. This rupture allows S-RNases to be released into the cytoplasm, where they can exert their cytotoxic effects. This process appears to be tightly coordinated with other pistil-side factors (Table 1). One such factor is stigma-expressed protein (NaStEP), a Kunitz-type protease inhibitor that functions in close proximity to HT-B. NaStEP is induced by self-pollination in *N. alata*, taken up into pollen tubes, and shown to be essential for maintaining SI (Jiménez-Durán et al. 2012; Cruz-Zamora et al. 2020). Silencing *NaStEP* results in destabilization of HT-B and the consequent loss of the SI response, highlighting their interdependent roles in promoting pollen rejection (Jiménez-Durán et al. 2012). Emerging evidence also implicates mitochondrial dysfunction as a downstream consequence of this pathway. The self-incompatibility pollen protein (NaSIPP), a mitochondrial transporter expressed in mature pollen, has been shown to interact in vivo with NaStEP within the mitochondria. This interaction increases mitochondrial membrane permeability, which is associated with the arrest of pollen tube growth and suggests that mitochondria may act as effectors in the terminal execution of the SI response (García-Valencia et al. 2017; Cruz-Zamora et al. 2020) (Table 2). Redox regulation has also been implicated in this model. NaTrxh directly binds and reduces S-RNases by targeting disulfide bonds, thereby enhancing their enzymatic activity (Torres-Rodríguez et al. 2020) (Table 1). Importantly, redox-inactive *NaTrxh* mutants are unable to support the SI response, reinforcing the importance of redox-mediated modulation of cytotoxicity during self-pollen rejection (Torres-Rodríguez et al. 2020).

Despite the growing body of evidence, several unresolved questions remain. Notably, the regulatory mechanisms underlying HT-B stabilization or degradation are not fully understood, and the subcellular dynamics of S-RNase localization continue to be debated. Adding further complexity, recent findings have shown that cytoskeletal disorganization occurs early during the SI response. Specifically, internalized S-RNases have been observed to disrupt cytoskeletal organization following endocytic uptake, and this disruption of the F-actin cytoskeleton has been shown to precede vacuolar membrane breakdown (Roldán et al. 2012). These results challenge the original framework of the compartmentalization model, in which the cytotoxic effect of S-RNases is thought to depend on vacuolar rupture. Thus, it is possible that a more complex interplay between S-RNase localization, cytoskeletal integrity, and signaling cascades occurs than previously appreciated, highlighting the need for continued investigation into the cellular basis of SI.

#### Other non-*S*-locus encoded factors involved in S-RNase-based SI

Following self-/non-self-recognition, divergence in pollen tube fate is primarily determined by the ability to sustain tip growth. While compatible pollen tubes elongate toward the ovule and achieve fertilization, self-pollen tubes rapidly arrest their growth. This outcome reflects the action of a complex intracellular network that extends beyond the well-characterized roles of degradation, condensation, and compartmentalization. In particular, studies in Rosaceae and Solanaceae have provided additional insights into how known cellular processes contribute to the arrest of self-pollen tubes (Table 2).

Among these processes, the actin cytoskeleton, which has already been implicated in both models, has emerged as a central downstream target of S-RNase cytotoxicity. The eukaryotic elongation factor 1A (eEF1A) was first identified from the pollen of *S. chacoense* as a protein that binds S-RNase (Soulard et al. 2014). The direct interaction between S-RNase and eEF1A enhances eEF1A binding to actin, thereby disrupting actin dynamics and ultimately arresting pollen tube growth. Similar mechanisms have been observed in *M. domestica*, S-RNase interacts with actin-severing proteins containing myosin villin and GRAM (MVG) during early pollination, disrupting actin filament turnover and impairing cytoskeletal homeostasis, thereby slowing tube growth (Yang et al. 2018). In addition to this indirect regulation, direct binding of S-RNase to Actin1 has also been observed in the pollen tubes of *Py. bretschneideri*. This interaction leads to severing of F-actin filaments and collapse of the cytoskeleton, which in turn initiates PCD (Chen et al. 2018). By contrast, the pollen-specific phospholipase D δ isoform 1 (PLDδ1) inhibits actin depolymerization triggered by S-RNase and directly interacts with Actin1 early in the SI response through its catalytic product, phosphatidic acid (Chen et al. 2018).

S-RNases also modulate calcium signaling in the pollen tube tip. In *Pyrus*, S-RNase entry into the pollen tube suppresses extracellular Ca^2+^ influx before RNA degradation is detectable. During this early stage, expression of calcium signaling-related genes is also downregulated suggesting that calcium signaling is directly impacted by S-RNase activity (Qu et al. 2016). Supporting this, in the pollen tubes of *Py. pyrifolia*, self S-RNase can inhibit Ca^2+^ influx by selectively reducing phospholipase C (PLC) activity in the plasma membrane (Qu et al. 2017). Downstream of this effect, a calcineurin B subunit protein5 (CBL5), highly expressed in apple pollen tubes, is critical for maintaining Ca^2+^ homeostasis (Gu et al. 2015). In vitro treatment of pollen tubes with an antisense oligonucleotide of *MdCBL5* inhibits their growth and reduces Ca^2+^ concentration. Moreover, S-RNase treatment can inhibit *MdCBL5* expression, indicating a regulatory cascade in which S-RNases manipulate intracellular Ca^2+^ levels to control pollen tube development. Closely intertwined with these events is redox homeostasis. In incompatible pollen tubes, S-RNase suppresses tip-localized ROS production in mitochondria and the apoplast, leading to a decrease in Ca^2+^ concentration and promotion of actin filament depolymerization. These events ultimately result in nuclear fragmentation and PCD (Wang et al. 2010; Claessen et al. 2019).

Besides cytoskeletal and ion signaling effects, S-RNases can also influence pollen tube wall remodeling. A recent study in *Pe. bretschneideri* uncovered that acetylation of inorganic pyrophosphatase 5 (PPA5) can be induced by S-RNases, which in turn repress the expression of the *pectin methylesterase44* (*PME44*) gene. The resulting impairment in cell wall remodeling leads to tip swelling in self-pollen tubes, another hallmark of growth arrest (Tang et al. 2023). Protein translation is another process disrupted by S-RNase. In *M. domestica*, interaction with soluble inorganic pyrophosphatase impairs tRNA aminoacylation in self-pollen tubes. Inhibition of this critical step in protein synthesis ultimately halts pollen tube elongation (Li et al. 2018). Finally, self-pollen rejection culminates in PCD, and several downstream markers of this process have been linked to S-RNase cytotoxicity. Repression of the V-ATPase subunit a1 by self S-RNase may contribute to this process (Kong et al. 2021). In pear and tobacco pollen tubes, suppression of this subunit correlates with decreased abundance and intracellular accumulation of diacylglycerol kinase 4, leading to vacuolar alterations and nuclear DNA degradation (Kong et al. 2021; Scholz et al. 2022). Together, these findings highlight that S-RNases act as multifaceted regulators, targeting not only nucleic acids but also key structural, signaling, and metabolic pathways to orchestrate the rapid arrest of self-pollen tubes.

#### Origins and evolution of diverse SI systems

The evolution of SI in angiosperms reflects a dynamic interplay between genetic innovation, lineage-specific adaptation, and genomic reorganization. Phylogenomic studies across diverse taxa indicate that Type-1 SI represents the ancestral system, likely originating in the core eudicot ancestor (Xue et al. 1996; Igic and Kohn 2001; Steinbachs and Holsinger 2002; Vieira et al. 2008; Ramanauskas and Igić 2017; Liang et al. 2020; Zhao et al. 2022). This system arose following the gamma whole-genome triplication (WGT) (∼120 My), which facilitated the physical linkage of ancestral T2 RNase (Class I) genes with F-box-associated (FBA/FBK) genes, forming a proto-*S*-locus (Liu et al. 2025). Functional assays support the existence of a broadly active ancestral SI system: SLFs from distantly related species such as Rosaceae and Ranunculaceae can effectively detoxify S-RNases from *Petunia* (Solanaceae), suggesting that early SLFs had wide substrate specificity before lineage-specific refinement (Zhao et al. 2022).

**Figure 5.**
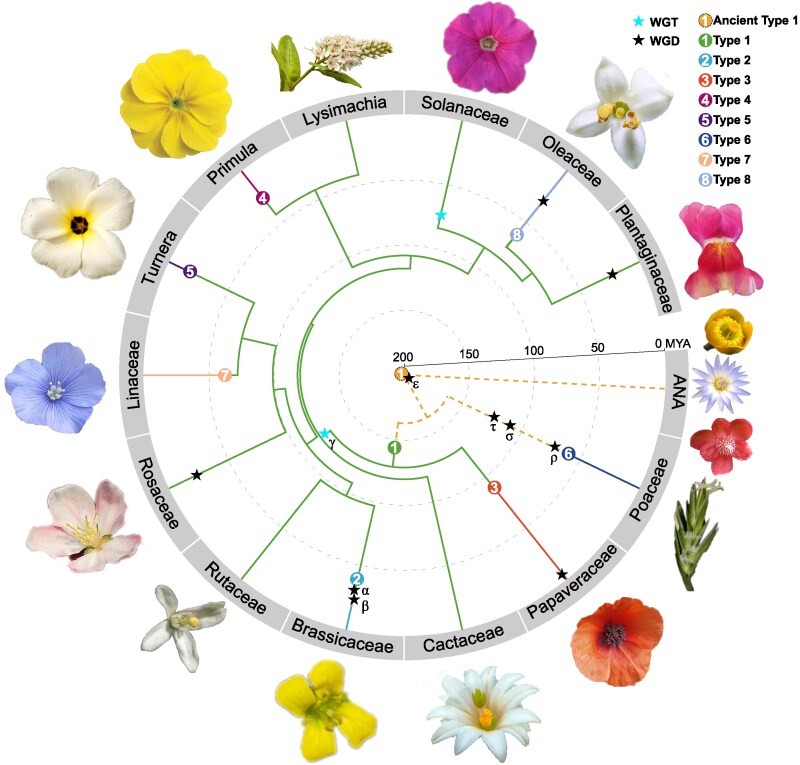
Phylogenetic distribution and evolutionary origins of SI systems in angiosperms. Circular cladograms show representative angiosperm families, with branch lengths scaled to divergence times (millions of years ago [MYA]). Eight distinct molecular SI mechanisms are mapped as colored numerals at the tips (see legend inset). “ANA” refers to the 3 earliest-diverging lineages (Amborellales, Nymphaeales, and Austrobaileyales). Tip labels (gray sectors) indicate family names. Floral photographs of each family were primarily obtained from Wikipedia (https://www.wikipedia.org/), except for the following: Nymphaeales (https://africawild-forum.com), Poaceae (https://powo.science.kew.org), Solanaceae (https://ballseed.com), Oleaceae (https://monaconatureencyclopedia.com), and Plantaginaceae (https://prairieseedshop.ca). Time estimates were obtained from TimeTree (https://timetree.org/), and the concentric circles mark 50 Myr intervals.

Type-1 SI has been retained in major clades of Plantaginaceae, Solanaceae, Rosaceae, Rutaceae, Cactaceae (Ramanauskas and Igić 2021), and *Lysimachia* (Ramanauskas et al. 2025) primarily through selective retention of the *S*-locus post whole-genome duplication (WGD), often via deletion of duplicate loci. Nonetheless, many lineages have lost Type-1 SI through *S-RNase* deletion (e.g. *A. majus*), pseudogenization (e.g. *Citrus*), or WGD-induced competitive interactions. Interestingly, SI has been regained in several lineages through the evolution of novel systems: Brassicaceae developed Type-2 SI after losing Type-1; Papaveraceae evolved Type-3 SI; *Primula* (Type-4), *Turnera* (Type-5), Linaceae (Type-7), and Oleaceae (Type-8) independently acquired sporophytic heterostyly (i.e. Type-4, -5, and -7) or either heterostyly or homomorphic SI (i.e. Type-8); and Poaceae evolved Type-6 SI involving *S* and *Z* loci (Zhao et al. 2022; Wang et al. 2025) (Fig. 5) (Jiao et al. 2011; Li et al. 2019; Su et al. 2021; Huang et al. 2023; Zheng et al. 2024; Jang et al. 2025).

In Plantaginaceae, dynamic changes such as deletion, expansion, and pseudogenization of ancestral SLFs further underscore the *S*-locus's evolutionary plasticity, as exemplified by the structurally complex snapdragon *S*-locus supergene (Zhu et al. 2023). Transposable elements have further contributed to *S*-locus diversification by facilitating rearrangements and transpositions of S-RNase/SLF clusters, particularly in Rosaceae and Solanaceae (Liu et al. 2025). Collectively, these patterns highlight the evolutionary plasticity of SI systems: ancestral genetic modules have been coopted, lost, or repurposed under shifting selective pressures, producing the diverse SI mechanisms observed in modern flowering plants.

## Perspectives

### Molecular basis of *S*-specificity

Comparative evolutionary analyses reveal that SLF alleles exhibit significantly lower nonsynonymous substitution rates compared to S-RNases across genera like *Antirrhinum*, *Petunia*, and *Prunus*, indicating a coevolutionary dynamic where novel S-RNases may temporarily evade SLF recognition, compelling compensatory adaptations in SLFs to maintain SI (Harkness and Brandvain 2021). This equilibrium faces tension due to the expanding diversity of S-RNases versus the constrained SLF repertoire, and in *Petunia*, for instance, a single population may host 9 to 27 *S*-haplotypes (Maenosono et al. 2024), generating over 1,000 combinations of SLF–S-RNase interactions, far exceeding traditional analytical capacities. Advances in genome assembly and tools like AlphaFold3 have enabled large-scale cataloging of proteins encoded by various *S*-locus haplotypes and structural prediction of protein interactions (Abramson et al. 2024). However, challenges persist: AlphaFold3 struggles with dynamic, context-dependent interactions, posttranslational modifications, and predicting high-confidence complexes for many allele pairs. To bridge this gap, integrative strategies merging AI-driven insights with sequence-based approaches are critical. Such efforts aim to resolve the molecular logic of *S*-specificity, addressing the paradox of maintaining robust discrimination amid allelic diversity shaped by balancing selection. By reconciling in silico predictions with in vivo outcomes, this integrative framework could unlock the molecular basis governing *S*-specificity.

### Genomic mechanisms of *S*-diversity generation

Recent advances in genome assembly have transformed the study of *S*-loci, offering a scalable strategy for identifying *S*-determinants through homology-based screening followed by functional validation. While systems like Papaveraceae GSI, Brassicaceae SSI, and grass family SI exhibit fixed *S*-gene numbers (2 or 3) within compact loci, S-RNase-based GSI systems display remarkable structural diversity, with loci spanning from ∼198 kb to ∼17 Mb, accompanied by variable SLF/SFB gene counts (9 to 37 genes) (Williams et al. 2014; Kubo et al. 2015; Li and Chetelat 2015; Li et al. 2019; Liang et al. 2020; Zhu et al. 2023; Gu et al. 2025). Nevertheless, challenges persist in assembling complex *S*-loci, particularly in Solanaceae, due to repetitive sequences and heterozygous phasing difficulties. Additionally, understanding the roles of colocalized “hitchhiking” genes and intergenic sequences within *S*-loci remains critical. High-quality assemblies also enable comparative evolutionary studies, revealing distinct *S*-locus localization patterns, pericentromeric in Solanaceae versus subtelomeric in Rutaceae and Rosaceae, reflecting divergent evolutionary trajectories of S-RNase-based systems. Future progress depends on ultra-long read sequencing and advanced bioinformatics to resolve complex *S*-loci, bridging structural, functional, and evolutionary insights to advance systems-level understanding of SI diversification.

### Possible mechanism of ancient Type-1 SI

A key open question is how a single-locus SI system could originate in the first place. It suggests that ancestral SLF proteins had broad detoxification capacities. In *Petunia*, SLFs from distantly related species could disable non-self S-RNases with high efficiency (Zhao et al. 2022). In other words, even an ancestral pistil RNase might have been “detoxified” by many SLFs, giving a high probability of successful non-self-fertilization. This promiscuous interaction would facilitate cross-pollination when only one SLF was present. Over time, additional SLFs could evolve, refining the system so that only self S-RNases escape detoxification. Evolutionarily, a high detoxification probability could explain the rapid spread of Type-1 SI: early plants benefited from many viable crosses, while eventually evolving multiple SLFs for stricter self-recognition. Further work (e.g. ancestral protein reconstructions) could test this scenario by measuring how ancestral SLFs and S-RNases interacted.

### Molecular dissection of additional SI mechanisms

SI is prevalent in Asteraceae and Fabaceae (legumes), which rank as the 2nd and 3rd largest angiosperm families, respectively. Despite its prevalence, the molecular basis of SI in these groups remains poorly understood. In Asteraceae, early evidence suggests an SSI system (Hughes and Babcock 1950). Genetic mapping has identified candidate intervals in sunflower, chicory, and *Silphium integrifolium*, with *Ha7650b* and *MDIS1-INTERACTING RECEPTOR LIKE KINASE2* (*MIK2*) proposed as candidate genes in sunflower and chicory, respectively (Badouin et al. 2021; Price et al. 2022; Palumbo et al. 2023). However, these candidates have yet to be functionally validated, requiring the continued advancement of transformation technologies.

In Fabaceae, SI mechanisms appear even more complex, occurring across diverse developmental stages, from stigma-level pollen rejection to postzygotic failure (Delaney and Igić 2022). In *Phaseolus vulgaris*, 2 type 1 *S*-like loci were identified, which may contribute to SC via competitive interactions (Zhao et al. 2022). Studies in *Trifolium repens*, *Medicago truncatula*, and *Cicer arietinum* have not identified pistil-specific T2-RNases, further diverging from the classical GSI model (Aguiar et al. 2015). Recent transcriptomic analyses in *M. sativa* point to a postzygotic SI response involving a broad array of regulatory genes (Li et al. 2024). Collectively, these findings challenge the applicability of classical SI genetic models to Fabaceae and highlight the need for systematic functional and evolutionary investigations to uncover their underlying molecular mechanisms.

### Connections among outcrossing mechanisms

Despite their differences, diverse outcrossing systems share common themes. All SI and heterostyly mechanisms ultimately enforce outcrossing by creating self-pollen–stigma incompatibility. Many involve tightly linked “supergenes” that coevolve to maintain genotype recognition. Frequent WGD events repeatedly break and reshape these supergenes (Zhu et al. 2023). Evolutionarily, the trend is dynamic: SI systems are easily lost when inbreeding is advantageous, but new mechanisms often evolve under selection for diversity (Zhao et al. 2022; Wang et al. 2025). In the future, comparative genomics across angiosperms may reveal whether certain gene families are repeatedly coopted for outcrossing, and how SI, heterostyly, and even dioecy intersect in the broader evolution of plant mating strategies.

### Application of SI in crop breeding

SI has been successfully utilized in agricultural breeding to develop hybrid varieties with enhanced vigor, yield, and adaptability. For example, breeders use SI lines in crops such as cabbage, radish, and other cruciferous plants to promote natural cross-pollination (Lao et al. 2014). This approach eliminates the need for labor-intensive manual emasculation and ensures the production of high-quality hybrid seeds. By selecting and maintaining self-incompatible parental lines, breeders can efficiently create hybrid populations that exhibit heterosis, or hybrid vigor, resulting in improved agronomic traits like larger fruit size, disease resistance, and uniformity. Moreover, advances in understanding the molecular and genetic basis of additional SI systems allow precise manipulation of this trait through modern biotechnological tools. This broadens the application of SI across diverse crop species, optimizing breeding workflows to advance sustainable agriculture by enabling the development of genetically diverse, high-yielding, and climate-resilient cultivars to meet global food security challenges.

## Data Availability

The data supporting the findings of this study are all available in the article.

## Dive Curated Terms

The following phenotypic, genotypic, and functional terms are of significance to the work described in this paper:

CBL5 Gramene: AT4G01420
CBL5 Araport: AT4G01420

SCR Gramene: AT3G54220
SCR Araport: AT3G54220

pollen AmiGo: PO:0025281

pme44 Gramene: AT4G33220
pme44 Araport: AT4G33220

PPa5 Gramene: AT4G01480
PPa5 Araport: AT4G01480

CUL1 Gramene: AT4G02570
CUL1 Araport: AT4G02570
