# Correction to: S-RNase-based self-incompatibility in angiosperms: Degradation, condensation, and evolution

**DOI:** 10.1093/plphys/kiag283

**Published:** 2026-06-10

This is a correction to: Yongbiao Xue, S-RNase-based self-incompatibility in angiosperms: Degradation, condensation, and evolution, *Plant Physiology*, Volume 199, Issue 1, September 2025, kiaf360, https://doi.org/10.1093/plphys/kiaf360

In the originally published version of this manuscript, in Figures 3 and 4, the proteins labeled S3-SLF in the Self Pollen Tube (S1) panels should instead be labeled S1-SLF. Figures 3 and 4 have been corrected.

Two typographical errors have also been corrected. ‘Targets’ in the abstract has been corrected to ‘target’ and ‘SRC: S-RNase Condensate’ has been added to the end of the legend for Figure 3.

